# Incorporation of spatial- and connectivity-based cortical brain region information in regularized regression: Application to Human Connectome Project data

**DOI:** 10.3389/fnins.2022.957282

**Published:** 2022-09-28

**Authors:** Aleksandra Steiner, Kausar Abbas, Damian Brzyski, Kewin Pączek, Timothy W. Randolph, Joaquín Goñi, Jaroslaw Harezlak

**Affiliations:** ^1^Department of Mathematics, Institute of Mathematics, University of Wroclaw, Wroclaw, Poland; ^2^Purdue Institute for Integrative Neuroscience, Purdue University, West Lafayette, IN, United States; ^3^School of Industrial Engineering, Purdue University, West Lafayette, IN, United States; ^4^Centre for Quantitative Research in Political Science, Jagiellonian University, Krakow, Poland; ^5^Faculty of Mathematics and Computer Science, Institute of Mathematics, Jagiellonian University, Kraków, Poland; ^6^Clinical Research Division and Public Health Sciences Division, Fred Hutchinson Cancer Research Center, Seattle, WA, United States; ^7^Purdue Institute for Integrative Neuroscience, Purdue University, West Lafayette, IN, United States; ^8^School of Industrial Engineering, Purdue University, West Lafayette, IN, United States; ^9^Weldon School of Biomedical Engineering, Purdue University, West Lafayette, IN, United States; ^10^Department of Epidemiology and Biostatistics, Indiana University School of Public Health - Bloomington, Bloomington, IN, United States

**Keywords:** linear regression, regularization, brain cortex, geodesic distance, euclidean distance, structural connectivity, HCP data, vocabulary comprehension

## Abstract

Studying the association of the brain's structure and function with neurocognitive outcomes requires a comprehensive analysis that combines different sources of information from a number of brain-imaging modalities. Recently developed regularization methods provide a novel approach using information about brain structure to improve the estimation of coefficients in the linear regression models. Our proposed method, which is a special case of the Tikhonov regularization, incorporates structural connectivity derived with Diffusion Weighted Imaging and cortical distance information in the penalty term. Corresponding to previously developed methods that inform the estimation of the regression coefficients, we incorporate additional information *via* a Laplacian matrix based on the proximity measure on the cortical surface. Our contribution consists of constructing a principled formulation of the penalty term and testing the performance of the proposed approach *via* extensive simulation studies and a brain-imaging application. The penalty term is constructed as a weighted combination of structural connectivity and proximity between cortical areas. Simulation studies mimic the real brain-imaging settings. We apply our approach to the study of data collected in the Human Connectome Project, where the cortical properties of the left hemisphere are found to be associated with vocabulary comprehension.

## 1. Introduction

Essential effort in neuroimaging studies has been devoted to examining the associations between the structure and the function of the brain. From the perspective of both medical applications and biostatistical analyses, it is still not a straightforward task to investigate the intricacies of brain structure's influence on cognitive functions. Use of different sources of information from the brain can be divided into parts, but the mutuality of some functions and structures is then ignored. In the context of the complex brain structure and its functionality, incorporating different types of data concurrently in the statistical method can be a more comprehensive approach to study cognitive functions. Therefore, in our work, we discuss the impact of the cortical distance on the regression parameter estimation.

The regularization method of ridgified Partially Empirical Eigenvectors for Regression (*riPEER*), described in Karas et al. (2019), uses linear regression to examine the relationship between brain structure and a scalar response. It is based on obtaining structural connectivity measurements from the cortex and applying them as information for the estimation of the regression coefficients. In Karas et al. (2019), as well as in this work, the structural connectivity measures are derived using Diffusion Weighted Imaging (DWI) processing pipeline which was comprehensively described in Ramírez-Toraño et al. (2021). In our study, we employ a different source of information, which informs the estimation of the model coefficients. From the biological point of view, there are substantial reasons to factor in the cortical regions' spatial distance.

Our approach is based on the multiple linear regression model:


(1) $$\begin{array}{l} y=X\beta +Zb+\varepsilon , \end{array}$$

where *y* is the vector of scalar responses for each of *n* individuals, *X* is the matrix of *n* observations with *m* predictors and their corresponding vector of unpenalized coefficients β∈ℝ^*m*^, and *Z* is an *n* × *p* matrix of variables with associated penalized coefficients in *p*-element vector *b*. Errors are stored in vector ε, where *E*(ε_*i*_) = 0 and $Var\left(\varepsilon_{i}\right)=\sigma^{2}$ for *i*∈{1, …, *n*}. The optimization problem that we want to focus on takes the form of *Tikhonov regularization*


(2) $$\begin{array}{l} \left[\begin{array}{c} \hat{b}\\ \hat{\beta } \end{array}\right]:=\underset{b,\beta }{\text{argmin}}\left\{||y-X\beta -Zb|{|}_{2}^{2}+b^{T}\left(\lambda_{C}{\tilde{Q}}_{C}+\lambda_{D}{\tilde{Q}}_{D}+\lambda_{R}I\right)b\right\}, \end{array}$$

with the penalty parameters λ_*C*_, λ_*D*_, λ_*R*_, and the corresponding penalty matrices ${\tilde{Q}}_{C}$ (connectivity-informed), ${\tilde{Q}}_{D}$ (distance-informed), *I* (ridge), respectively.

We consider the spatial proximity of the regions in the brain cortex. The spatial distance between the nodes can be defined both as a Euclidean distance and as a geodesic distance. The latter, when defined on the cortex, refers to the shortest path along the cortical surface (Oligschläger et al., 2017). The Euclidean-based distances were retrieved using the centroids data from the HCP repository. The geodesic distance measurements for this work were calculated by Dr. Abbas using Python packages surfdist and nibabel. In our investigation, the definition and understanding of the spatial proximities in the human brain cortex is based on the work undertaken by Yamin et al. (2019), where the nodes' proximity is defined as a function of spatial distance between them. The measure of similarity is then used as a counterpart of proximity. These measures are incorporated in the normalized Laplacian of the distance matrix (Reinhart, 2021) to define the penalties.

Incorporating structure *via* a matrix in the regularization term is not new and can be found in the literature (see Engl et al., 1996; Bertero et al., 2001; Benning and Burger, 2018). This matrix can be used to include elements of structural information between the predictor variables in the model. Commonly used matrices are for instance the second-difference matrices that impose smoothness on the estimates. The literature (Hastie et al., 1995) shows that imposing spatial smoothness constraints on the coefficients is efficient and sometimes essential in terms of both prediction performance and interpretability. In our prior publication (Karas et al., 2019) utilizing the structural connectivity measures obtained with DWI processing as prior information, we summarized an extensive simulation study where we studied different forms of the matrix Q. The other example of this is a structural connectivity between the brain nodes as in Karas et al. (2019) and Brzyski et al. (2021), which is defined as a strength of the connections between the gray matter regions *via* the density of the structural white matter fibers (Hagmann et al., 2008). In statistics, it is often presented in the form of the adjacency matrix (or so-called connectivity matrix), a well-known concept in graph theory (Harary, 1962). The graph Laplacian of the adjacency matrix is often used as a “smoothing penalty” (Reinhart, 2021). Extension of the penalty term with the cortical areas' connectivity information was proposed by Karas et al. (2019).

The framework for incorporating auxiliary information to improve the regression coefficient estimation is based on well-established statistical concepts. First is the Tikhonov regularization method, which considers the optimization problem with a symmetric semi-positive definite matrix Q in the additional term λ*b*^*T*^Qb added to the residual sum of squares Example of Tikhonov regularization is *ridge regression*, which penalizes squares of the model's coefficients (hence, the penalty matrix *Q* is an identity matrix). Furthermore, the *PEER* (Partially Empirical Eigenvectors for Regression) approach (Randolph et al., 2012), a special case of the Tikhonov “generalized ridge,” employs a scientific-information-based matrix *Q* and a penalty parameter λ as the regularization term.

A crucial component of any penalized regression problem is the selection of parameters that ultimately define the regression estimate in Equation (2). Cross-validation is one common procedure used to estimate these parameters (see Brezinski et al., 2003; Lukas, 2006). An alternative is Restricted Likelihood Estimation (REML) (Corbeil and Searle, 1976; Maldonado, 2005), whose properties in this context were discussed in detail by Reiss and Todd Ogden (2009). Although in this work, the information incorporated into the optimization problem comes from two different sources, the main emphasis is put on the proximity derived from the distance. The connectivity-based information has been comprehensively discussed and developed in the previous work of the riPEER method in Karas et al. (2019).

The remainder of this article is organized as follows. Section 2 introduces the theory behind our proposed *disPEER* (distance-based *PEER*) method. We present the settings and the results of the simulation study in Section 3, and the discussion is presented in Section 4.

## 2. Materials and methods

### 2.1. Statistical model and optimization

Let us consider the setting where we have *n* observations of a random variable in vector *y* and two design matrices, where *n* rows denote the number of observations. The columns of the first design matrix, *X*∈ℝ_*n* × *m*_ denote the variables that do not correspond to the cortex itself, and their coefficients will not be penalized. In the second design matrix *Z*∈ℝ_*n* × *p*_, columns represent the variables for which there is information about the structural connectivity and the spatial proximity. The *p* × 1 vector of regression coefficients *b*, is penalized in the optimization problem.

The information about structural connectivity and spatial proximity given for the covariates in matrix *Z* is contained in so-called adjacency matrices A_*C*_ and $A_{\tilde{D}}$, respectively. We assume that Equation (1) represents the model equation.

In this work, analogously to Karas et al. (2019), we use the equivalence of our regularization setting with the Linear Mixed Model formulation, where $b\sim N\left(0,\sigma_{b}^{2}Q^{-1}\right)$ with $Q=\lambda_{C}{\tilde{Q}}_{C}+\lambda_{D}{\tilde{Q}}_{D}+\lambda_{R}I$. It can be treated as a link between the optimization problem in Equation (2) and a Bayesian prior assumption about the coefficients' distribution as in Maldonado (2005). The addition of a small parameter λ_*R*_ with an identity matrix is a ridge-like parameter that accounts for the possible non-invertibility of Q and enables the maximum likelihood estimation of regularization parameters—this approach, called AIM (Adding Identity Matrix), comes from Karas et al. (2019). The approach here is that Q is merely “close” to the true signal matrix ℚ. As mentioned in Brzyski et al. (2021), an assumption is that information encoded by the adjacency matrix is a general approximation of the real brain structure rather than representing the actual structure of this complex human organ. The penalty Laplacian matrix ${\tilde{Q}}_{C}$ that will be considered in this study is based on the structural connectivity and it is estimated using DWI sequence.

For clarity and convenience, we include all the notation used in the article in Table 1.

**Table 1:** Notation used in the manuscript.

**Notation**	**Description**
*c*_*ij*_	Euclidean distance between *x*_*i*_ and *x*_*j*_
*d*_*ij*_	Spatial distance between nodes *i* and *j*
${\tilde{d}}_{ij}$	Normalized spatial distance between nodes *i* and *j*
*a*_*ij*_	Spatial proximity between nodes *i* and *j*
*n*	Number of observations
*p*	Number of penalized variables
*m*	Number of unpenalized variables
*y*	Vector of the responses
ε	Vector of random errors
*w*_*i*_	Degree of the *i*−*th* row of A_*C*_
*s*_*i*_	Degree of the *i*−*th* row of A_*D*_
*X*	*n* × *m* design matrix
*Z*	*n* × *p* design matrix
β	*m*-element vector of unpenalized fixed effects
*b*	*p*-element vector of penalized coefficients
A _*C*_	Adjacency matrix based on the structural connectivity
A _*D*_	Adjacency (proximity) matrix based on the distance
${\tilde{Q}}_{C}$	Normalized Laplacian based on the structural connectivity
${\tilde{Q}}_{D}$	Normalized Laplacian based on the spatial proximity

### 2.2. Distance-based penalties

In the penalization problem in Equation (2), we partition the overall design matrix into two distinct design matrices *X* and *Z*, where the coefficients of the latter are penalized. As in Karas et al. (2019) and Brzyski et al. (2021), we add a penalty term connected only with the variables describing the cortex, e.g., the variables that contain structural information.

The *riPEER* (Karas et al., 2019) approach incorporated brain connectivity-based information in the penalty term. Our aim is to extend it by additionally incorporating spatial-distance-based information in the penalty terms. We study the estimation procedures, wherein the regression coefficients corresponding to the cortical measurements (in the *riPEER* example—average thickness values) contain in some way not only the information about the structural connectivity but also information obtained from the distance between brain regions. We formally introduce this concept, referred to as *disPEER* (distance-based Partially Empirical Eigenvectors for Regression), in the following sections. In order to do so, we define and elaborate on the concepts of distance and its principled incorporation in the penalty terms. In our work, we use a common cortex parcellation approach called the Desikan-Killiany parcellation (Desikan et al., 2006), which partitions the human cortical surface into 68 regions.

**Approach to distance term in the cortex**. The purpose of this work is to study regularization methods for regression that are informed not only by the structural connectivity between the cortical areas but also by the spatial distance between them.

**Figure 1:**
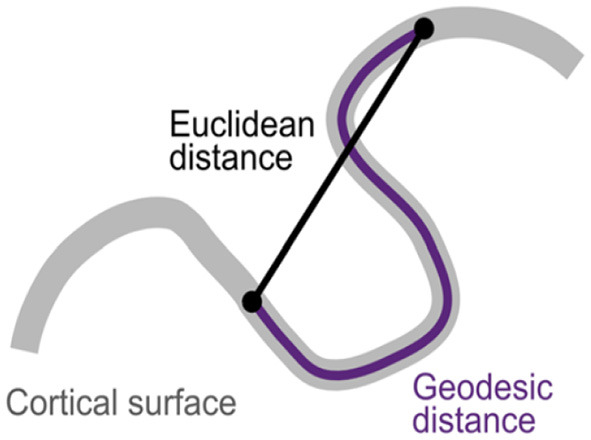
Geodesic distance in contrast to Euclidean distance. Geodesic distance between two points is the shortest distance along the cortical surface. The image comes from Oligschläger et al. (2017).

The structure of the human brain, however, implies that the Euclidean distance between nodes does not accurately reflect their proximity within the cortex. A more appropriate measure of distance should account for the folded structure of the cortex, with its grooves and peaks (see Figure 1). To account for this, beyond the Euclidean distance, we adopt also the geodesic distance (Yamin et al., 2019) along the cortical surface to measure proximities between two nodes. This is conveniently provided by the computational tool FreeSurfer^1^. Parcellation term here refers to a particular definition of a specific cortical division.

Denote by *d*_*ij*_ the geodesic distance between nodes *i* and *j*, and let D = [*d*_*ij*_] be the *p* × *p* matrix of all such distances. Then D is a symmetric matrix with non-negative entries and zeros on the diagonal. In order to manage discrepancies in human brain sizes, we normalize distances in *D* to the interval [0, 1]. Because we want to avoid the situation where our distance is equal to 0 when in practice it is not, we use the following normalization


(3) $$\begin{array}{l} {\tilde{d}}_{ij}=\frac{d_{ij}}{\underset{0\leq i,j\leq p}{max}d_{ij}}. \end{array}$$

The resulting matrix $\tilde{D}=\left[{\tilde{d}}_{ij}\right]$ has entries expressing distances transformed into the interval [0, 1] and will be the basis for defining the proximity matrix used in our penalty function.

**Transforming distance into proximity**. Let us define the matrix $A_{\tilde{D}}=A_{{\tilde{D}}_{ij}},1\leq i,j\leq p$, which is an adjacency matrix. It is a symmetric matrix with non-negative elements and zeros on the diagonal. The adjacency concept in this matrix is represented by the physical closeness and the idea behind it is to determine physical closeness based on the spatial distance between the brain regions. When we consider the adjacency matrix defined by the connections between the nodes, a larger number indicates a stronger connection. In adjacency defined by physical closeness, a larger number means a smaller distance. Once we get the normalized distance matrix $\tilde{D}$ we clarify the definition of the adjacency matrix A.

We employ the measure of similarity, which is commonly used in techniques for nonlinear dimension reduction (Trosset, 2021). Then, the adjacency matrix is defined by:


(4) $$A_{{\tilde{D}}_{ij}}:\;=\{\begin{array}{ll} \exp(-h{\tilde{d}}_{ij}^{2})\text{, for some }h>0, & \text{if }i\neq j\\ {\tilde{d}}_{ij}, & \text{otherwise}. \end{array}$$

Due to the *h* parameter in Equation (4), we can adjust the values of proximity and spread them between 0 and 1 in that manner so the order of magnitude of the maximum and minimum adjacency ratio is proximate to some target value. This is useful when we want to put more emphasis on the regions that are really close by in comparison to the regions that are far apart.

**Laplacian matrix**. In defining the optimization problem for the estimation of parameters, we will use the concept of adding structural information into the method of regularization. This structural information will be given by a Laplacian matrix originating in the proximity (adjacency) matrix. Therefore, assuming the already defined adjacency matrix $A_{\tilde{D}}$ in Equation (4), the element *a*_*ij*_ denotes the spatial proximity between the nodes *i* and *j*. Denote the *degree* of node *i* by $s_{i}:=\overset{p}{\underset{j=1}{\sum }}a_{ij}$; i.e., the sum of all proximities with node *i* and let *S*: = *diag*(*s*_1_, …, *s*_*p*_). The Laplacian of the adjacency matrix $A_{\tilde{D}}$ is $Q_{\tilde{D}}=S-A_{\tilde{D}}$.

The normalized Laplacian ${\tilde{Q}}_{\tilde{D}}$=$\left[{\tilde{Q}}_{D_{ij}}\right]$, 1≥*i, j*≥*p* for the proximity matrix based on the distance is defined as:


(5) $${\tilde{Q}}_{{\tilde{D}}_{ij}}:\;=\{\begin{array}{ll} -A_{{\tilde{D}}_{ij}}/\sqrt{s_{i}s_{j}}, & \text{if }i\neq j\\ 1, & \text{otherwise}, \end{array}$$

where *s*_*i*_ is the degree of *i*^*th*^ row of adjacency-proximity matrix $A_{{\tilde{D}}_{ij}}$. Observe that a penalty of the form $b^{T}{\tilde{Q}}_{{\tilde{D}}_{ij}}b=\overset{p}{\underset{i,j=1}{\sum }}A_{{\tilde{D}}_{ij}}{\left(b_{i}-b_{j}\right)}^{2}$ implies that the greater the proximity is between two nodes, the greater the penalty is on the (squared) difference between their corresponding coefficients.

### 2.3. Estimation

When formulating the optimization problem in Equation (2), we include two versions of a Laplacian matrix, Q_*C*_ and $Q_{\tilde{D}}$. The first one is based on the structural connectivity, and the second one has origins in the spatial distance. Knowing these two definitions, one can observe the specific structure for the matrices containing structural connectivity and spatial distance. Matrix A_*C*_, which represents the connections between the nodes, will be naturally sparse (as there are no direct connections between many cortical regions) and the distance matrix $A_{\tilde{D}}$ will be dense (as none of the distance matrix entries will be equal to zero, as these entries represent distances between the distinct brain regions).

The maximum likelihood estimates of the penalty parameters and error variance are


(6) $$\{\begin{array}{l} {\hat{\text{λ}}}^{MLE}=\underset{\left({\text{λ}}_{C},{\text{λ}}_{D},{\text{λ}}_{R}\right)}{\text{argmin}}\tilde{l}({\text{λ}}_{C},{\text{λ}}_{D},{\text{λ}}_{R})\\ {\hat{\sigma }}_{\varepsilon }^{2MLE}\;=\;\frac{1}{n}y_{P}^{T}{\left(\tilde{Z}B_{\text{λ}}\tilde{Z}T+I_{n}\right)}^{-1}y_{P} \end{array},$$

where $\tilde{l}$ is the linear mixed model log-likelihood function of λ_*C*_, λ_*D*_, and λ_*R*_, $y_{P}=\tilde{Z}b+\tilde{\varepsilon }$, $\tilde{Z}=P_{X}^{C}Z$ with $P_{X}^{C}:=I_{n}-X{\left(X^{T}X\right)}^{-1}X^{T}$ and $B_{\lambda }:=\lambda_{C}{\tilde{Q}}_{C}+\lambda_{D}{\tilde{Q}}_{D}+\lambda_{R}I$. The detailed derivation of the formulas in Equation (6) is in the Supplementary material.

Further, we numerically solve the optimization problem from Equation (2) and find estimates of λ_*C*_, λ_*D*_ and λ_*R*_ according to Equation (6). Therefore, we were able to determine the estimates of the *b* and β coefficients according to the equations:


(7) $$\begin{array}{l} {\hat{b}}^{new}:={\left({\tilde{Z}}^{T}\tilde{Z}+{\hat{\lambda }}_{C}Q_{C}+{\hat{\lambda }}_{D}Q_{D}+{\hat{\lambda }}_{R}I\right)}^{-1}{\tilde{Z}}^{T}y_{P} \end{array}$$

and


(8) $$\begin{array}{l} {\hat{\beta }}^{new}:={\left(X^{T}X\right)}^{-1}X^{T}\left(y-Z{\hat{b}}^{new}\right). \end{array}$$

Naturally, when calculating the coefficients' estimates using Equation (7), we take into account the observed Laplacian Q_*C*_ and true Laplacian matrix based on the geodesic distance. We assume that the latter reflects the real state almost perfectly, because of the fact that with today's tools, it is possible to measure the distance between cortex regions quite precisely. However, the other aspect is that in practice we do not know the exact *h* parameter that proximity is determined by. Hence, we check the impact of this uncertainty in further numerical experiments.

We have now defined all the components of the optimization problem in Equation (2) and are ready to employ the *disPEER* approach. The following sections summarize the simulation study and the HCP data application, where we compare the performance of *disPEER* with *riPEER, Ridge* regression, and ordinary least squares. In the simulation part, we investigate the behavior of the considered methods in the situations where distance has an impact on the estimation. Further, in Section 3.3, we examine the estimation performance of the aforementioned methods on the brain data obtained from the Human Connectome Project repository. Preparation of the data and implementation of the statistical methods provided in R scripts can be found online^2^.

## 3. Results

### 3.1. Simulation study

We conducted numerical experiments to study the estimation accuracy where the information sources contain both the connectivity between the cortical regions and their spatial proximity.

#### 3.1.1. Simulation settings

**Figure 2:**
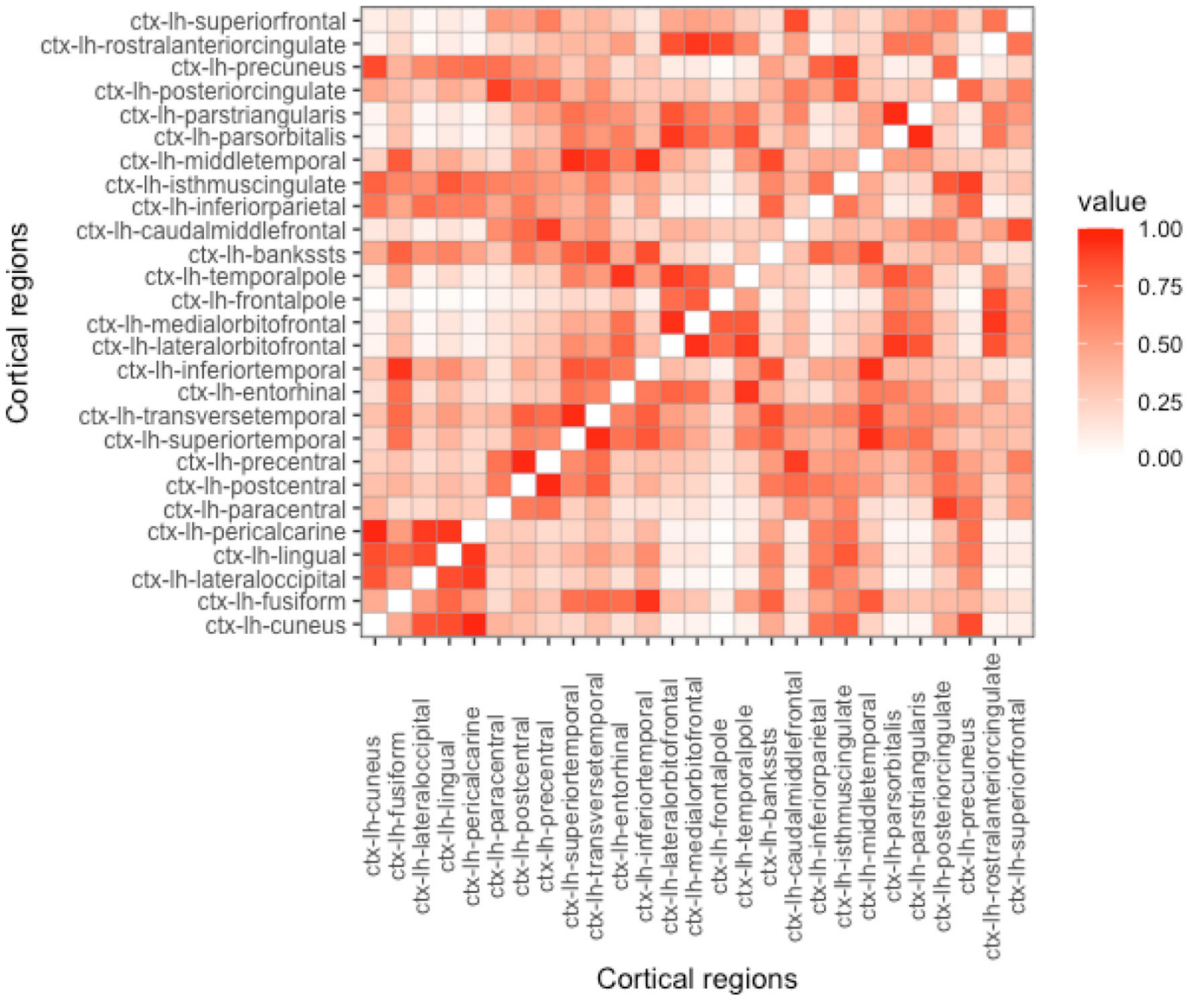
Proximity matrix displayed for the left hemisphere, Euclidean distance, and parameter *h* = 5. See other considered *h*-based proximity matrices in the Supplementary material.

**Figure 3:**
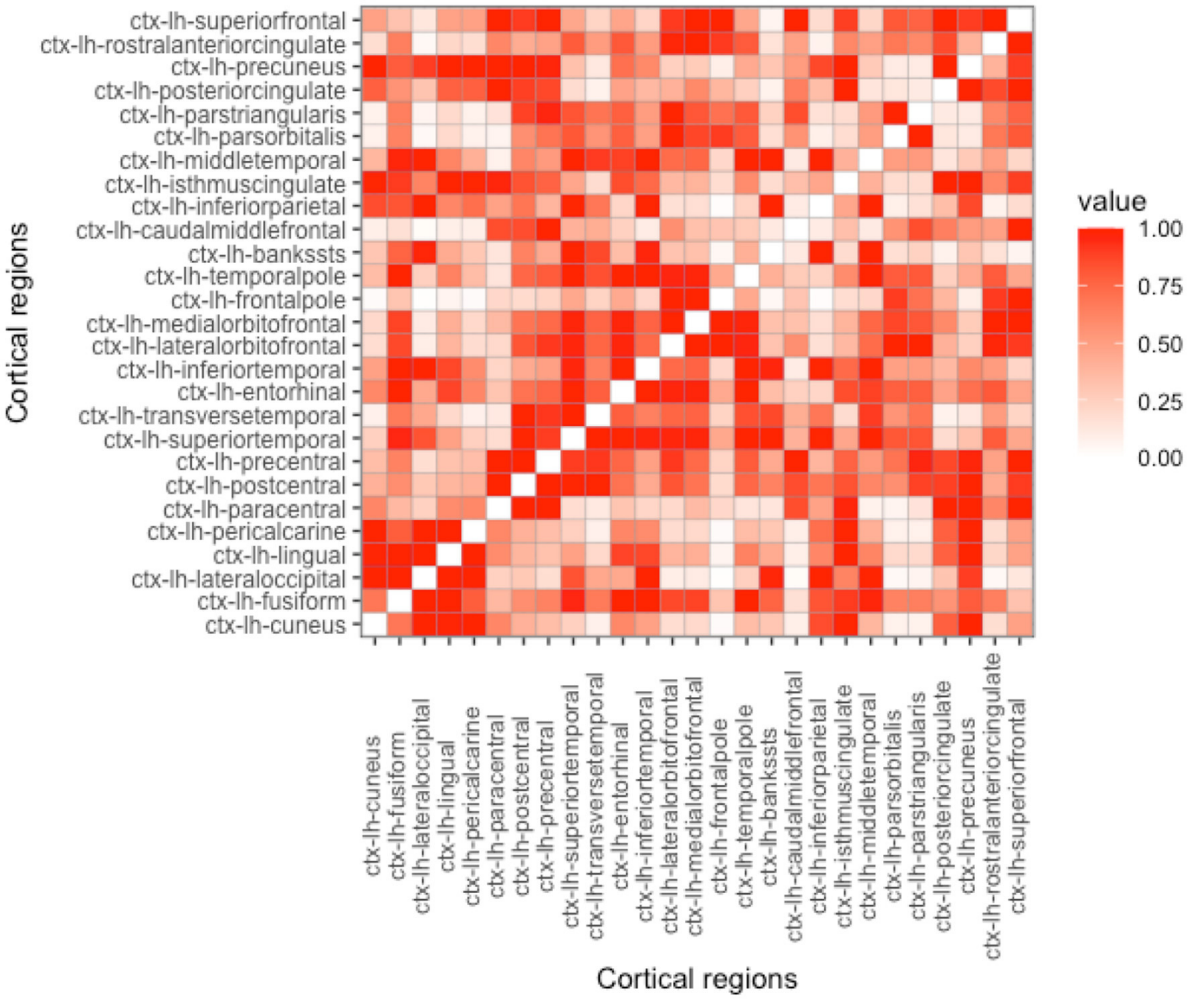
Proximity matrix displayed for the left hemisphere, geodesic distance, and parameter *h* = 5. See other considered *h*-based proximity matrices in the Supplementary material.

**Figure 4:**
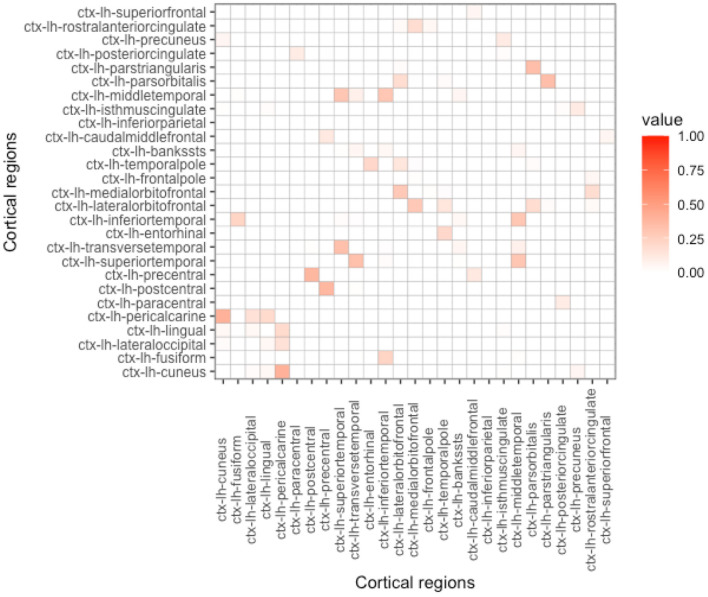
Proximity matrix displayed for the left hemisphere, Euclidean distance, and parameter *h* = 100. See other considered *h*-based proximity matrices in the Supplementary material.

**Figure 5:**
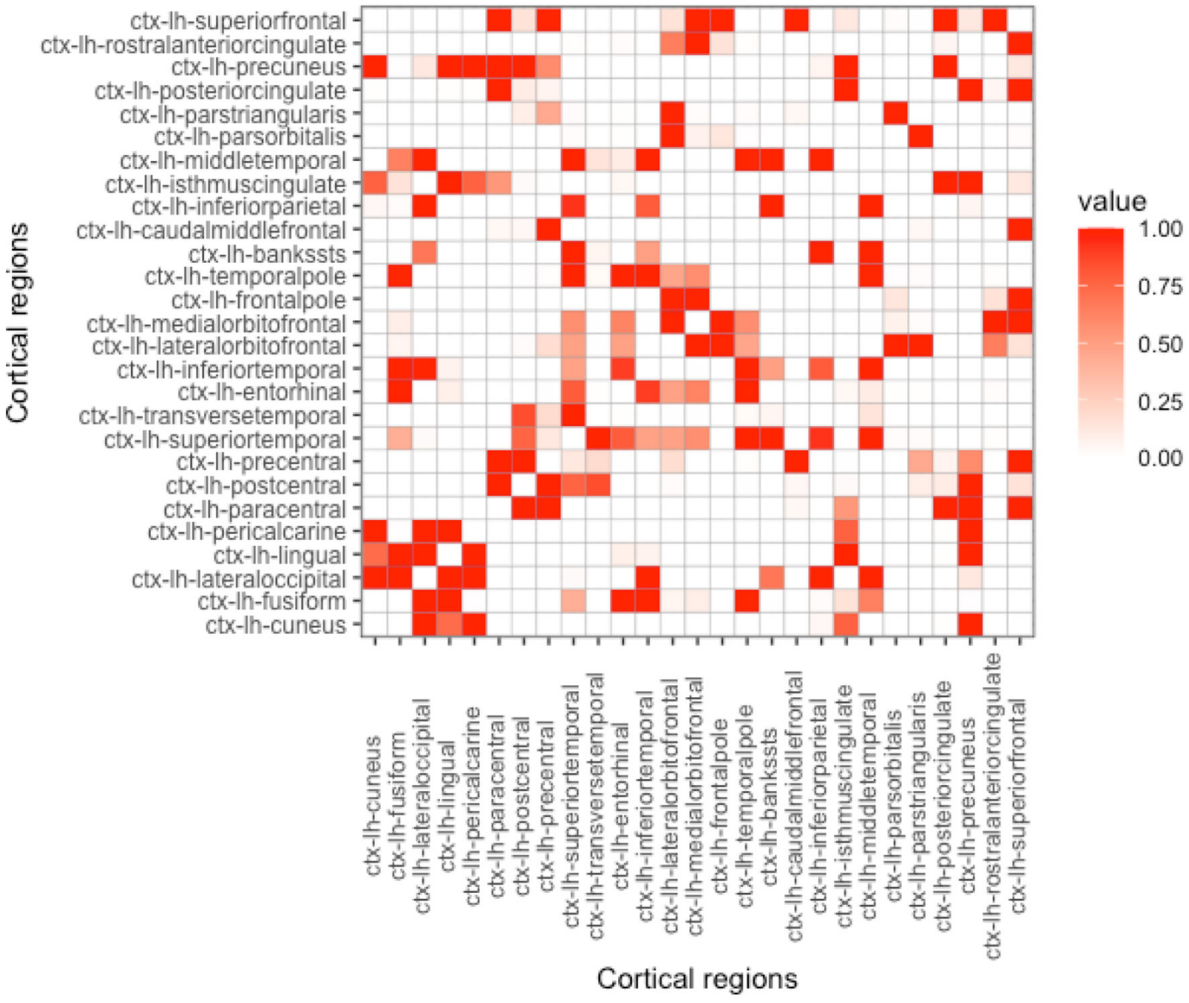
Proximity matrix displayed for the left hemisphere, geodesic distance, and parameter *h* = 100. See other considered *h*-based proximity matrices in the Supplementary material.

1. **Informativeness of the connectivity-adjacency matrix**
  Utilizing the setup of Karas et al. (2019), we assume that the connectivity information used for the estimation approximates the truth.
  In the process of generating data we use A_*C*_*true*__ and in the estimation, we use A_*C*_*obs*__ which partially contains the information from the true matrix. In order to apply this setting, let us use the measure of dissimilarity between A_*C*_*true*__ and A_*C*_*obs*__ from the *riPEER* paper:
  (9) $$\begin{array}{l} diss(A_{C_{obs}},A_{C_{true}})\\ =\frac{\sum_{1\leq i,j\leq p}1\{|A_{C_{obs}}[i,j]-A_{C_{true}}[i,j]|\;>0\}}{2\cdot \sum_{1\leq i,j\leq p}1\{A_{C_{true}}[i,j]>0\}}. \end{array}$$
2. **Informativeness of the proximity-adjacency matrix**
  Brain-imaging software currently available (e.g., FreeSurfer) allows us to measure the distances in the cortex accurately. Taking into account this fact, we can presume that the information content of the distance matrix is quite high.In our numerical experiments, we use a fixed value of the *h* parameter, which determines proximity measurements in the proximity-adjacency matrix (see Equation 4). In practice, we do not have this parameter at our disposal, as we do not know it. This is why we want to investigate *disPEER* performance, as well as putting approximate information in the proximity matrix.
3. **Information sources**
  We experimented with a number of assumptions on where the information for the regularization method is coming from. We want to study how both connectivity and spatial proximity have an impact on the estimation of the *b* coefficients. We studied these approaches by considering a few settings of the parameters *r*_*C*_ and *r*_*D*_ in the covariance matrix of *b*: *B*_*true*_ = *r*_*C*_Q_*C*_*true*__+*r*_*D*_Q_*D*_*true*__+*r*_*R*_*I*_*p*_, where Q_*C*_*true*__ and Q_*D*_*true*__ are the Laplacians of corresponding true adjacency matrices. In our setting, the relationship between *r*_*C*_ and *r*_*D*_ is that these parameters sum up to 1: *r*_*C*_ = 1−*r*_*D*_.
4. **Selection of the hemisphere**
  The two hemispheres of the human brain are connected *via* the *corpus callosum* which is not a part of the cortex. This is the reason why it is not possible to calculate the geodesic distance between the regions that are not in the same hemisphere. Of course, technically, we can determine the Euclidean distance through the two hemispheres. Nevertheless, a very natural way to determine distance *via* the brain cortex is geodesic distance. As the distances estimated using both Euclidean and geodesic definitions are highly correlated, we chose to present the majority of the results using the Euclidean distance. The corresponding results for the geodesic distance are qualitatively similar. Analyzing the associations in the two hemispheres separately could be a natural solution in this situation:
  (10) $$\left[\begin{array}{c} \hat{b}\\ \hat{\beta } \end{array}\right]:=\underset{b,\beta }{\text{argmin}}\{{\left\|y_{P}-\tilde{Z}b\right\|}_{2}^{2}+b^{T}({\text{λ}}_{C}^{H}{\tilde{Q}}_{C}^{H}+{\text{λ}}_{D}^{H}{\tilde{Q}}_{D}^{H}+{\text{λ}}_{R}^{H}I)b$$
  where *H* marks one of the hemispheres: left or right.
  When analyzing the data, we consider 27 and 29 regions in the left and the right hemisphere, respectively. To properly account for both sources of information, structural connectivity and cortical distances, and to illustrate our approach, we concentrated on the blocks with at least four brain regions. That is, we excluded from the analysis connected blocks of size three or less. The final regions' affiliation to the blocks was determined by the algorithm developed by Dr. Goñi in Ramírez-Toraño et al. (2021).
5. **Determining the proximity**
  As can be seen in Equation (4), proximity is determined by the parameter *h*. Depending on its value, the influence on the nearby regions in comparison to the regions that are far apart can be either emphasized or de-emphasized. With the increase of *h*, while the Euclidean-based proximity measure is shrunk in a semi-continuous way, geodesic-based proximity resembles a more binarized form (see Figures 2–5). In the simulation studies, our estimation procedure employs different values of the parameter *h* to study its influence on the regression coefficients' estimation error.

#### 3.1.2. Synthetic data generation

To set up the variables for further generating the response, we take the real adjacency matrix A_*C*_*true*__ and create A_*C*_*observed*__ fixing the established value of dissimilarity parameter *diss*(A_*C*_*obs*__, A_*C*_*true*__). From both of these matrices, we determine Laplacian matrices Q_*C*_*true*__ and Q_*C*_*observed*__. Also, for the distance matrix, we use the matrix of distances obtained in the Euclidean space. Next, we calculate the adjacency (proximity) matrix using parameter *h*, and based on that, we get the Laplacian Q_*D*_. With true matrices Q_*C*_*true*__ and Q_*D*_, we are able to determine the distribution of *b* coefficients in the data generation.

Initially, we specify several settings of the parameters:

- Number of observations *n* = 400
- Number of predictors *p*∈{29, 27} (depending on the hemisphere) and *m* = 2
- Signal strength $\sigma_{b}^{2}\in \left\{0.01,0.1\right\}$
- Variance of error $\sigma_{\varepsilon }^{2}\in \left\{1,5\right\}$ (having a situation with stronger and weaker signals from the data)
- Parameter of dissimilarity *diss*(A_*C*_*obs*__, A_*C*_*true*__) = 0.25
- Fraction of the information coming from the connectivity *r*_*C*_∈{0.1, 0.5, 0.9}, where part of the information coming from the spatial proximity is *r*_*D*_ = 1−*r*_*C*_

We generate synthetic data according the specification below:

1. Matrix *Z*∈ℝ^*n* × *p*^ with *iid* rows from N(**0**, *I*_*p*_)
2. Matrix *X*∈ℝ^*n* × *m*^ with *iid* rows from N(**0**, *I*_*m*_)
3. Coefficients vector $b\sim N\left(0,\sigma_{b}^{2}B_{true}^{-1}\right)$
4. Coefficients vector β = (0, 0)^*T*^
5. Response vector *y* = *Xβ*+*Zb*+ε with $\varepsilon \sim N\left(0,\sigma_{\varepsilon }^{2}I_{n}\right)$

The settings above are considered separately for each of the two hemispheres. For a particular hemisphere, we find corresponding Laplacians based either on the structural connectivity or the distance. As in Karas et al. (2019), to evaluate the estimation accuracy, we study the relative mean squared error, which is expressed as


(11) $$\begin{array}{l} \text{rMSE}\left(\hat{b}\right)=\frac{||\hat{b}-b|{|}_{2}^{2}}{||b|{|}_{2}^{2}}. \end{array}$$

### 3.2. Study of the estimation error and its components: Bias and variance

Mean Squared Error is the sum of the variance and squared bias. In addition to studying classical *MSE* measure of all the methods' estimates, we also examined its components to have a more comprehensive picture of the *disPEER* estimation quality, e.g., to see the relationship between the variance and squared bias.

**Figure 6:**
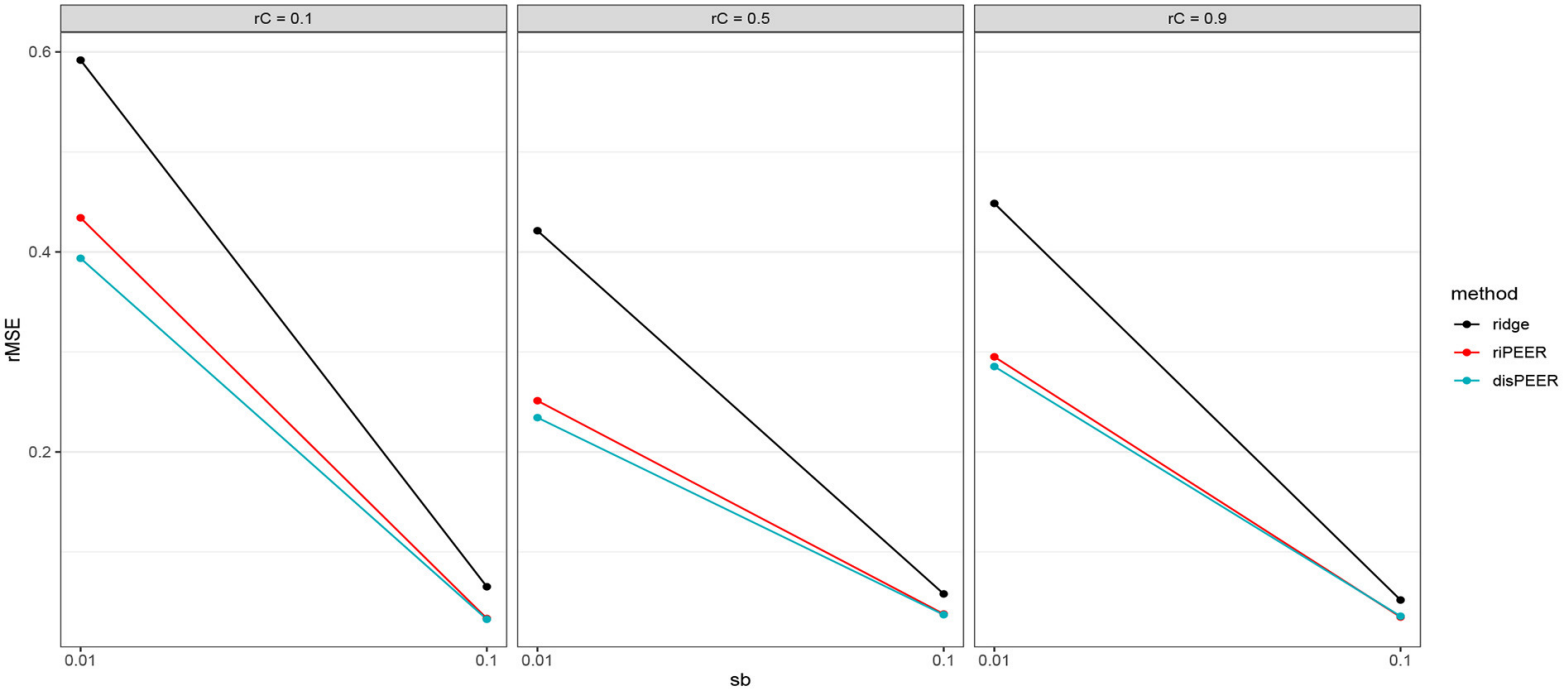
Plots of *rMSE* (medians) of *b* estimates for different connectivity information configurations *r*_*C*_∈{0.1, 0.5, 0.9} with σ_*b*_∈{0.01, 0.1}, σ_ε_ = 1, and *h* = 5 for *ridge, riPEER*, and *disPEER* (**left hemisphere** and **Euclidean distance**).

As Figure 6 shows, median *rMSE* of the *b* coefficient values estimates is lower for *disPEER* than either for *riPEER* or for *ridge regression*. It can also be seen from the results presented in Table 2 that for the majority of the settings *disPEER* shows the best performance. All these results are obtained under the assumption that we know the true value of the parameter *h* with which the proximity is determined. However, using different values of *h* in the estimation, we can still show better performance of our method (see Supplementary material). The results in Table 2 also show that the *disPEER* method works very well, especially for *r*_*C*_ = 0.1, independently of the distance approach used. It is an expected behavior, as it is the setting where our method is supposed to work the best, because the relationship among the coefficients *b* is mainly driven by the information coming from the distance matrix. We notice that the rMSE percentage decrease reaches up to 20% compared with the *riPEER* approach. Even if we equally distribute origination of information for the generation of the *b* coefficients between both connectivity- and distance-based information, *disPEER* still prevails over its sister method *riPEER*. We also conducted experiments to study the behavior of *rMSE* when *n*∈{100, 200} (Supplementary Tables 1, 2). In these settings, the information content is smaller relative to the sample size and there are cases when *ridge regression* provides smaller *rMSE* than brain-information-based methods. This behavior is especially visible in the situations when the ratio of $\sigma_{b}^{2}/\sigma_{\varepsilon }^{2}$ is the lowest, hence, the signal to noise ratio is small: $\sigma_{b}^{2}/\sigma_{\varepsilon }^{2}=0.002$. Moreover, the number of cases when *rMSE* for *riPEER* and *disPEER* that are close to each other increased in comparison to *n* = 400.

**Table 2:** *rMSE* (median) results for *ridge, riPEER*, and *disPEER* methods with *h* = 5 and *h* = 100 for Euclidean and geodesic distances, respectively, for $\sigma_{b}^{2}\in \left\{0.1,0.01\right\}$ and $\sigma_{\varepsilon }^{2}\in \left\{1,5\right\}$.

***rMSE*** **results (100 loops)**						
*r*_*C*_	$\sigma_{b}^{2}/\sigma_{\varepsilon }^{2}$	**Distance**	***Ridge***	***riPEER***	***disPEER***	***rMSE*** **Decrease [%]**
0.1	0.100	*Euc*.	0.0652	0.0334	0.0326	2.5
0.1	0.010	*Euc*.	0.5918	0.4341	0.3936	10.3
0.1	0.020	*Euc*.	0.3087	0.1483	0.1425	4.1
0.1	0.002	*Euc*.	0.9909	0.8179	0.7283	12.3
0.5	0.100	*Euc*.	0.0580	0.0379	0.0372	1.9
0.5	0.010	*Euc*.	0.4212	0.2512	0.2343	7.2
0.5	0.020	*Euc*.	0.2287	0.1539	0.1449	6.2
0.5	0.002	*Euc*.	0.9821	0.6612	0.5844	13.1
0.9	0.100	*Euc*.	0.0519	0.0349	0.0357	−2.2
0.9	0.010	*Euc*.	0.4485	0.2951	0.2854	3.4
0.9	0.020	*Euc*.	0.2403	0.1533	0.1518	1.0
0.9	0.002	*Euc*.	0.9831	0.8714	0.8902	−2.1
0.1	0.100	*geod*.	0.0729	0.0503	0.0487	3.3
0.1	0.010	*geod*.	0.4923	0.4236	0.3737	13.4
0.1	0.020	*geod*.	0.3143	0.2367	0.2175	8.8
0.1	0.002	*geod*.	0.9907	1.1820	0.9439	19.3
0.5	0.100	*geod*.	0.0723	0.0403	0.0398	1.3
0.5	0.010	*geod*.	0.5110	0.3323	0.3117	6.6
0.5	0.020	*geod*.	0.2765	0.2363	0.2226	6.2
0.5	0.002	*geod*.	0.9948	0.6137	0.6186	−0.8
0.9	0.100	*geod*.	0.0556	0.0561	0.0561	0.0
0.9	0.010	*geod*.	0.3588	0.1623	0.1634	−0.7
0.9	0.020	*geod*.	0.2148	0.2634	0.2618	1.0
0.9	0.002	*geod*.	0.9559	0.5286	0.5439	−2.8

Last column denotes percentage of *rMSE* decrease for *disPEER* in respect of *riPEER* (“–” indicates increase).

**Figure 7:**
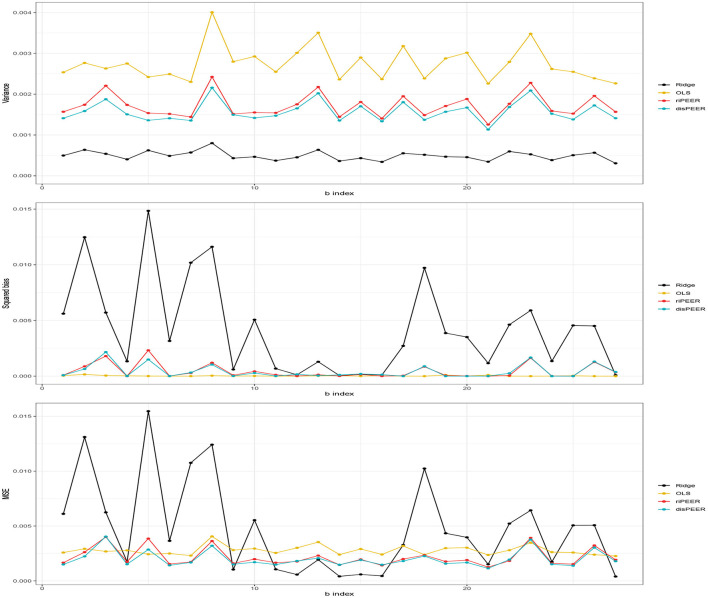
Empirical variance, squared bias, and MSE of *b* estimates for σ_*b*_ = 0.01, σ_ε_ = 1, *r*_*C*_ = 0.5, and *h* = 5 (**left hemisphere** and **Euclidean distance**).

Figure 7 shows that our approach helps in keeping the balance between the variance and squared bias trade-off in contrast to *ridge regression*, which despite showing very low variance cannot cope with prominently higher bias (hence, worse *MSE*). Also, the distance-based approach has slightly lower variance than *riPEER*. We do not claim any theory-based properties here. However, our extensive simulation studies show that our new method, *disPEER*, shows improved performance when compared with other regularization methods. We cannot claim uniform improvement in every single case, but it is undoubtedly a prevalent phenomenon.

**Proximity parametrization—sensitivity analysis**. In Table 2, we present the results for the true values of the proximity parameters *h* equal to 5 and 100 for Euclidean and geodesic distances, respectively. We chose these values, because we want to keep the distance information we give for coefficient estimation as useful as possible. In Figures 2, 4, one can observe that *h* should not be too large in the Euclidean approach, because more and more regions become close to each other and we lose the differentiation in the proximity matrix. In contrast, what happens with geodesic distance (Figures 3, 5) is that with the increase of *h*, we observe that proximities get decidedly more binarized. Our Supplementary Figure 2 shows that our estimation procedure is robust to the mis-specification of the parameter *h*.

### 3.3. Human Connectome Project data application

We want to compare the estimation methods presented in Section 2, especially the new method *disPEER* in the context of real data. Our aim here is to consider the numerical response, which is a particular measure of cognition. All analyses are adjusted for the demographic data (gender and age) with their coefficients unpenalized (fixed effects). Regression coefficients associated with the cortical volume are penalized (random effects). To perform this analysis, we will use the data from the Human Connectome Project repository (HCP). Also, we will use the data on the brain anatomic measurements: structural connectivity and brain cortical regions' spatial proximities. These data are not available in the HCP repository. To obtain these measurements, we used the pipeline developed and implemented by Dr. Goñi (Ramírez-Toraño et al., 2021).

#### 3.3.1. Data description and preparation

As a part of the application of the studied methods on the imaging data, we used the publicly available data from the Human Connectome Project. This is a project funded by the National Institutes of Health HCP with the original goal of building a map of the neural connections and producing the data in order to study brain disorders. The HCP repository contains data on 1,200 young adults in the age range of 22–35. The data we use do not contain all the 1,200 subjects from the HCP study. Initially, we chose adults in such a way that we do not have related persons among them, so we analyzed only independent observations. It led to data from 428 unrelated individuals. Cortical properties and the cortical regions' coordinates in the HCP study were obtained using the FreeSurfer software.

We focus on both the cortical thickness and cortical area data as in further work we consider the volume which is the product of the thickness and the area. We found a few outliers in both hemispheres and we decided to replace their data with their family members' data or, in cases where this was not possible, we deleted such data from our analysis. As a result, we used 424 and 426 subjects in the analysis of the left and right hemispheres, respectively.

#### 3.3.2. HCP data analysis

To achieve a meaningful interpretation of the *disPEER*'s performance in the case of real data, we wanted to compare it with other statistical methods. We studied the estimation using *Ridge Regression, Ordinary Least Squares* assuming a linear regression model with coefficients [β, *b*], the *riPEER* method with fixed and random effects and a penalty term containing the Laplacian originating from the structural connectivity, and our proposed *disPEER* approach, also with a linear mixed model formulation and penalty component incorporating the structural connectivity and spatial proximity information. In the HCP data setting, the coefficients of *Gender* (categorical 0/1 variable) and *Age* (numerical variable), corresponding to X matrix, are not penalized. We penalize the coefficients of individual-brain-normalized cortical volume measurements (*Z* matrix), which are obtained by multiplying the average cortical thickness and cortical areas. We normalize regions' volumes by dividing every measurement by the subject's cortical volume in the particular hemisphere. This is because brain volumes especially differ between men and women; on average, men's brain have higher volumes than women's. As a response *y*, we chose the measurements of Language/Vocabulary Comprehension, which measures a participant's receptive vocabulary. The respondent is presented with an audio recording of a word and four images on the computer screen and is asked to select the picture that most closely matches the meaning of the word. In the estimation process, we used standardized variables (mean 0 and variance 1) in the *X* and *Z* matrices and outcome vector *y*. For *riPEER* we applied *R* package *mdpeer* with connectivity-based penalty-Laplacian ${\tilde{Q}}_{C}$. With the usage of this matrix and additionally a proximity Laplacian ${\tilde{Q}}_{D}$, we estimated model coefficients with *disPEER*.

#### 3.3.3. Estimation of the coefficients for language/vocabulary comprehension

**Figure 8:**
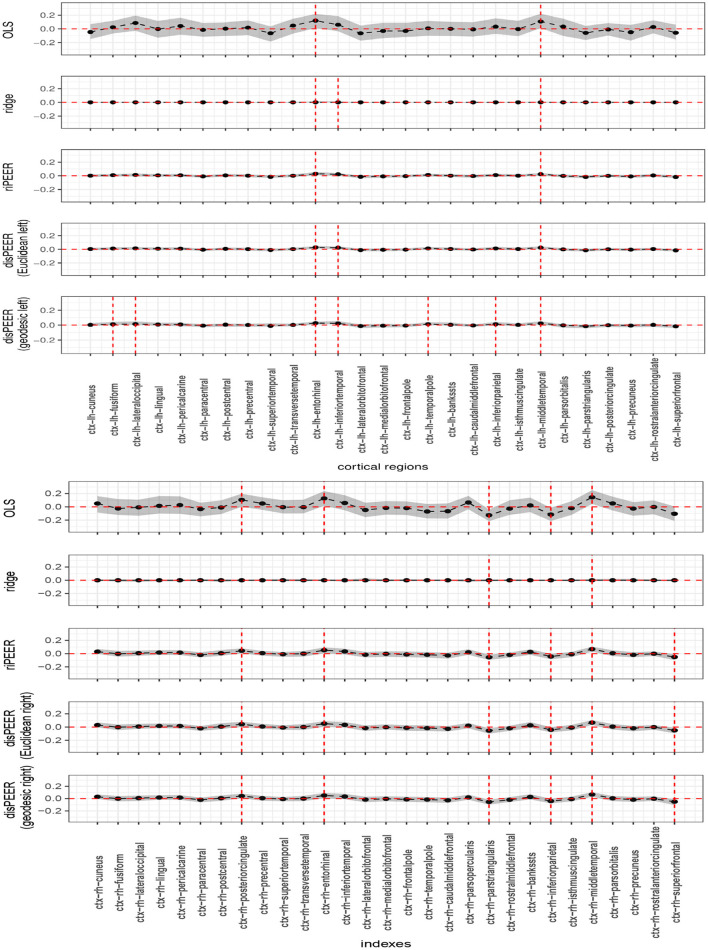
Estimates of *b* for Language/Vocabulary Comprehension cognitive function with non-adjusted 95% bootstrap-based confidence intervals denoted for left and right hemisphere (**top** and **bottom**, respectively). Results are presented as estimates (solid black lines), coefficient confidence intervals (shaded areas), zero line (horizontal red line), and significant coefficient at 95% level (vertical red lines).

Coefficient estimates obtained using *disPEER* for the left hemisphere turned out to be primarily driven by the proximity as was seen in the penalty parameter estimates. Coefficients indicated as significant in the right hemisphere are the same for both *disPEER* and *riPEER* Figure 8 which can be easily explained by the fact that, in this setting, our method is mainly driven by the connectivity information. Therefore, it is highly akin to the connectivity-based approach.

**Figure 9:**
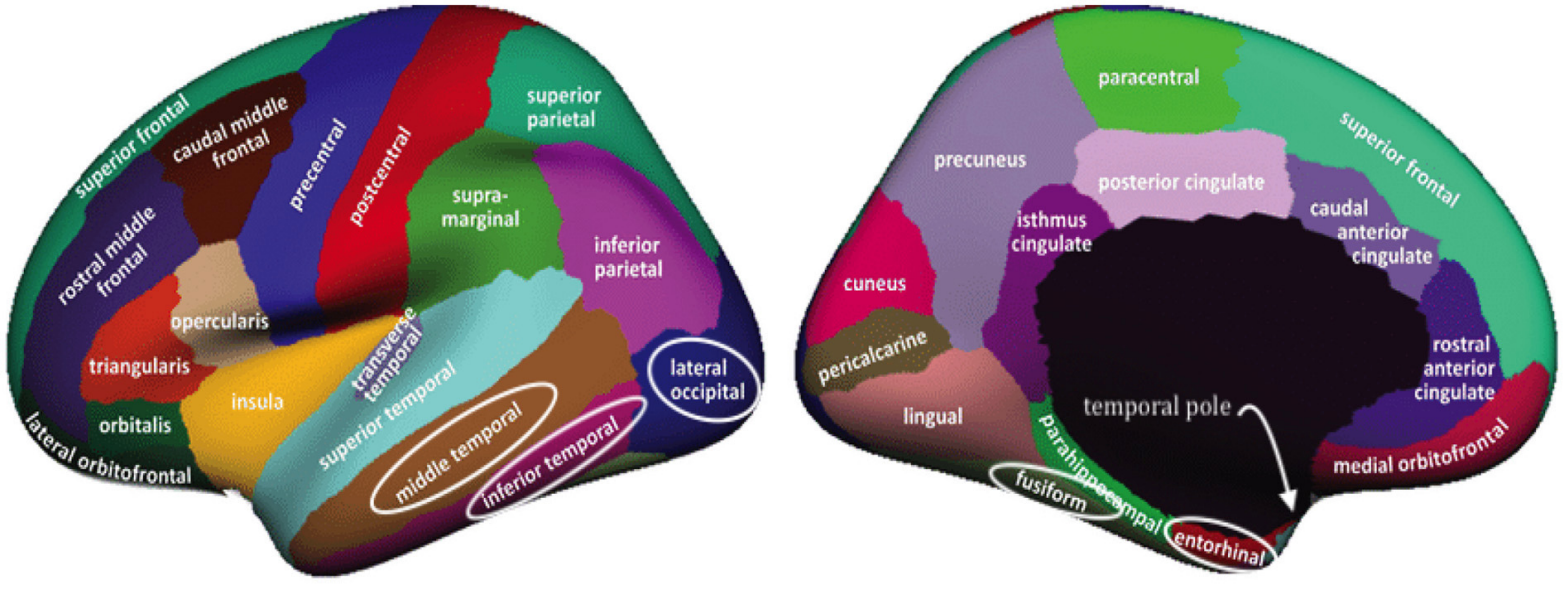
Desikan-Killiany parcellation (original image from Klein and Tourville, 2012) of the left hemisphere with significant regions indicated by white loops. Regions are visible from lateral and medial perspectives (**left** and **right**, respectively).

We indicated in the manuscript that the confidence intervals for each predictor are based on the univariable approximation using the bootstrap-based confidence intervals. *disPEER* estimates, when the geodesic distance is employed, imply the highest number of significant regions in relation to the other methods. Moreover, it turns out that the significantly associated areas are located close to each other on the cortical surface. Based on the *disPEER* estimates, we conclude that they may be involved in language comprehension. We marked these regions using white rectangles in Figure 9. Also, we summarized the significant regions in Table 3, where we included corresponding *p*-values computed for bootstrap results. Indeed, the literature (Blank et al., 2002; Fridriksson et al., 2015; Mesulam et al., 2015) states that some areas of the left hemisphere are associated with language comprehension. The so-called Wernicke's and Broca's areas are the two main zones commonly known for being associated with speech production. However, the location and functionality of these regions are still not fully established. In the literature, we also find many different studies that demonstrate that normal communicative speech is reliant on the left hemisphere regions that are distant from the classically defined language areas of Wernicke's and Broca's areas (Blank et al., 2002; Mesulam et al., 2015).

**Table 3:** Cortical regions in the left hemisphere with corresponding bootstrap-obtained *p*-values and non-adjusted 95% confidence intervals (for geodesic distance).

**Cortical region**	**Associated *p*-value**	**Associated confidence interval**
Inferior temporal	0.002	[0.01394, 0.05408]
Middle temporal	0.002	[0.01053, 0.05270]
Entorhinal	0.008	[0.00824, 0.04749]
Fusiform	0.029	[0.00183, 0.04251]
Lateral occipital	0.033	[0.00186, 0.04876]
Temporal pole	0.047	[0.00002, 0.04142]

## 4. Discussion

The complexity of the human brain was a primary factor driving the search for another source of information that could improve the study of the associations between the structural properties of the cortex and neurocognitive outcomes as manifested by improvement in the regression coefficient estimation. In our work, we provided an extension of the method developed in Karas et al. (2019) by incorporating a distance measure among cortical regions to provide additional information in the penalization specified in Equation (2).

We studied the performance of the coefficient estimation with the incorporation of the structural connectivity and spatial proximity, and as an evaluation measure, we examined the relative MSE of coefficients defined in Equation (11). In our simulation study, we used two types of the distance term—Euclidean and geodesic distance. Also, we assumed different impacts on model coefficients from both sources of information. We observed that independently of the parameter specification and assumptions we make for the distance or *b* distribution, in most cases, estimation benefits from the incorporation of the between-region distance information. There is a significant improvement in *disPEER* estimation in terms of not only *rMSE* but also bias. Regarding the bias-variance trade-off, it is clearly visible that while *ridge regression* has the smallest variance with the highest bias, at the same time, *disPEER* and *riPEER* keep balance between these measures, with a visible advantage of our distance-based method.

*disPEER* was applied to the real data collected by the Human Connectome Project. We studied a cognitive function of vocabulary comprehension and noticed that in the left hemisphere, *disPEER* estimation was driven mainly by the proximity and cortical regions chosen by this method were located adjacently. Moreover, these neighboring regions indicated as significant by *disPEER* were situated within the so-called Wernicke's and Broca's areas, which according to the literature are involved in language ability (see Blank et al., 2002; Fridriksson et al., 2015; Mesulam et al., 2015).

In our future work, we will explore utilizing sparsity-inducing penalties, e.g., LASSO (Tibshirani, 1996). We will also expand our predictor space to include the activation maps from the task-based fMRI studies with the penalties imposed by both spatial distance and structural connectivity matrices and we will incorporate the simulation-based approach of Ruppert et al. (2003) to establish simultaneous confidence intervals for all predictors.

## Data availability statement

Publicly available datasets were analyzed in this study. This data can be found at: http://www.humanconnectomeproject.org/data/.

## Ethics statement

Ethical review and approval was not required for the study on human participants in accordance with the local legislation and institutional requirements. The patients/participants provided their written informed consent to participate in this study.

## Author contributions

AS and JH implemented the statistical model and its application to the synthetic and real data and created the first draft of the manuscript. TR, DB, and KP contributed to the theoretical part of the study and provided feedback for the paper. JH, KA, and JG processed the cortical data including calculation of the geodesic and Euclidean distance. All authors contributed to the manuscript revisions, read, and approved the submitted version.

## Funding

This research was mainly conducted when AS was a Short-Term Visiting Research Scholar at the Indiana University School of Public Health-Bloomington on Exchange Visitor Program P-1-00104 in 2021. Partial support for this research was provided by the NIMH grant R01 MH108467 and by the NINDS grant R01 NS112303.

## Conflict of interest

The authors declare that the research was conducted in the absence of any commercial or financial relationships that could be construed as a potential conflict of interest.

## Publisher's note

All claims expressed in this article are solely those of the authors and do not necessarily represent those of their affiliated organizations, or those of the publisher, the editors and the reviewers. Any product that may be evaluated in this article, or claim that may be made by its manufacturer, is not guaranteed or endorsed by the publisher.

## Footnotes

^1^ https://surfer.nmr.mgh.harvard.edu

^2^ https://www.github.com/AleksandraSteiner/disPEER_data

## References

[B1] Benning M. Burger M. (2018). Modern regularization methods for inverse problems. Acta Numer. 27, 1–111. 10.1017/S0962492918000016

[B2] Bertero M. Boccacci P. Koenig A. (2001). Introduction to inverse problems in imaging. Opt. Photon. News 12, 46–47. 10.1201/9781003032755

[B3] Blank S. Scott S. Murphy K. Warburton E. Wise R. (2002). Speech production: Wernicke, Broca and beyond. Brain 125, 1829–1838. 10.1093/brain/awf191 12135973

[B4] Brezinski C. Redivo-Zaglia M. Rodriguez G. Seatzu S. (2003). Multi-parameter regularization techniques for ill-conditioned linear systems. Numer. Math. 94, 203–228. 10.1007/s00211-002-0435-8

[B5] Brzyski D. Karas M. M. Ances B. Dzemidzic M. Goni J. W. Randolph T.. (2021). Connectivity-informed adaptive regularization for generalized outcomes. Can. J. Stat. 49, 203–227. 10.1002/cjs.11606 35002039 PMC8730330

[B6] Corbeil R. R. Searle S. R. (1976). Restricted maximum likelihood (REML) estimation of variance components in the mixed model. Technometrics 18, 31–38. 10.2307/1267913

[B7] Desikan R. S. Ségonne F. Fischl B. Quinn B. T. Dickerson B. C. Blacker D.. (2006). An automated labeling system for subdividing the human cerebral cortex on MRI scans into gyral based regions of interest. NeuroImage 31, 968–980. 10.1016/j.neuroimage.2006.01.021 16530430

[B8] Engl H. W. Hanke M. Neubauer A. (1996). Regularization of Inverse Problems, Vol. 375. Dordrecht: Springer Science & Business Media. 10.1007/978-94-009-1740-8

[B9] Fridriksson J. Fillmore P. Guo D. Rorden C. (2015). Chronic Broca's aphasia is caused by damage to Broca's and Wernicke's areas. Cereb. Cortex 25, 4689–4696. 10.1093/cercor/bhu152 25016386 PMC4669036

[B10] Hagmann P. Cammoun L. Gigandet X. Meuli R. Honey C. J. Wedeen V. J.. (2008). Mapping the structural core of human cerebral cortex. PLoS Biol. 6:e159. 10.1371/journal.pbio.0060159 18597554 PMC2443193

[B11] Harary F. (1962). The determinant of the adjacency matrix of a graph. SIAM Rev. 4, 202–210. 10.1137/1004057

[B12] Hastie T. Buja A. Tibshirani R. (1995). Penalized discriminant analysis. Ann. Stat. 23, 73–102. 10.1214/aos/1176324456

[B13] Karas M. Brzyski D. Dzemidzic M. Goni J. Kareken D. A. Randolph T. W.. (2019). Brain connectivity-informed regularization methods for regression. Stat. Biosci. 11, 47–90. 10.1007/s12561-017-9208-x 31217828 PMC6583926

[B14] Klein A. Tourville J. (2012). 101 labeled brain images and a consistent human cortical labeling protocol. Front. Neurosci. 6:171. 10.3389/fnins.2012.00171 23227001 PMC3514540

[B15] Lukas M. A. (2006). Robust generalized cross-validation for choosing the regularization parameter. Inverse Probl. 22:1883. 10.1088/0266-5611/22/5/02112558252

[B16] Maldonado Y. M. (2005). Mixed Models, Posterior Means and Penalized Least Squares. College Station: Texas A&M University.

[B17] Mesulam M.-M. Thompson C. Weintraub S. Rogalski E. (2015). The wernicke conundrum and the anatomy of language comprehension in primary progressive aphasia. Brain 138, 2423–2437. 10.1093/brain/awv154 26112340 PMC4805066

[B18] Oligschläger S. Huntenburg J. M. Golchert J. Lauckner M. E. Bonnen T. Margulies D. S. (2017). Gradients of connectivity distance are anchored in primary cortex. Brain Struct. Funct. 222, 2173–2182. 10.1007/s00429-016-1333-7 27807628 PMC5504232

[B19] Ramíirez-Tora no F. Abbas K. Bru na R. Marcos de Pedro S. Gómez-Ruiz N. Barabash A.. (2021). A structural connectivity disruption one decade before the typical age for dementia: a study in healthy subjects with family history of Alzheimer's disease. Cereb. Cortex Commun. 2:tgab051. 10.1093/texcom/tgab051 34647029 PMC8501268

[B20] Randolph T. W. Harezlak J. Feng Z. (2012). Structured penalties for functional linear models-partially empirical eigenvectors for regression. Electron. J. Stat. 6:323. 10.1214/12-EJS676 22639702 PMC3358792

[B21] Reinhart C. (2021). The normalized distance Laplacian. Spec. Matrices 9, 1–18. 10.1515/spma-2020-0114

[B22] Reiss P. T. Todd Ogden R. (2009). Smoothing parameter selection for a class of semiparametric linear models. J. R. Stat. Soc. Ser. B 71, 505–523. 10.1111/j.1467-9868.2008.00695.x

[B23] Ruppert D. Wand M. P. Carroll R. J. (2003). “Semiparametric regression,” in Cambridge Series in Statistical and Probabilistic Mathematics (Cambridge: Cambridge University Press). 10.1017/CBO9780511755453

[B24] Tibshirani R. (1996). Regression shrinkage and selection *via* the lasso. J. R. Stat. Soc. Ser. B 58, 267–288. 10.1111/j.2517-6161.1996.tb02080.x

[B25] Tikhonov A. N. (1963). Solution of incorrectly formulated problems and the regularization method. Soviet Math. Dokl. 4, 1035–1038.

[B26] Trosset M. W. (2021). Proximity in Dimension Reduction, Clustering, and Classification. Available online at: https://mtrosset.pages.iu.edu/Courses/675/notes.pdf

[B27] Yamin A. Dayan M. Squarcina L. Brambilla P. Murino V. Diwadkar V.. (2019). “Comparison of brain connectomes using geodesic distance on manifold: a twins study,” in 2019 IEEE 16th International Symposium on Biomedical Imaging (ISBI 2019) (Venice), 1797–1800. 10.1109/ISBI.2019.8759407

