# Supplementary material for: Incorporation of spatial- and connectivity-based cortical brain region information in regularized regression: Application to Human Connectome Project data
Source: Front Neurosci. 2022 Sep 28;16:957282. doi: 10.3389/fnins.2022.957282 (PMC9555077; doi:10.3389/fnins.2022.957282)
Supplement: Supplementary file 1 [file Data_Sheet_1.pdf]

Supplementary Material

1 ESTIMATION OF THE MODEL PARAMETERS

According to the convex optimization problem of the Tikhonov regularization we consider optimization problem with the extension of the penalty term:

$$\begin{bmatrix} \hat{b} \\ \hat{\beta} \end{bmatrix} := \underset{b,\beta}{\operatorname{argmin}}\{||y - Zb - X\beta||_2^2 + b^T(\lambda_C \tilde{\mathcal{Q}}_C + \lambda_D \tilde{\mathcal{Q}}_D + \lambda_R I)b\}. \tag{S1}$$

The reason for including $b^T \lambda_R b$ in the problem S1 is that we want $B_\lambda := \lambda_C \tilde{\mathcal{Q}}_C + \lambda_D \tilde{\mathcal{Q}}_D + \lambda_R I$ to be invertible.

Considering projection $\mathcal{P}_X^C := I_n - X(X^T X)^{-1} X^T$, the problem defined in S1 can be rewritten as

$$\underset{b}{\operatorname{argmin}}\{||\mathcal{P}_X^C y - \mathcal{P}_X^C Zb||_2^2 + b^T B_\lambda b\} \tag{S2}$$

It is easy to notice that

$$\mathcal{P}_X^C y = \mathcal{P}_X^C (Zb + X\beta + \varepsilon) = \mathcal{P}_X^C Zb + \mathcal{P}_X^C \varepsilon.$$

Therefore, assuming that $y_\mathcal{P} := P_X^C y$, $\tilde{Z} := P_X^C Z$ and $\tilde{\varepsilon} := P_X^C \varepsilon$ we can finally consider the model

$$y_\mathcal{P} = \tilde{Z} b + \tilde{\varepsilon} \tag{S3}$$

and reformulate the optimization problem as:

$$\begin{bmatrix} \hat{b} \\ \hat{\beta} \end{bmatrix} := \underset{b,\beta}{\operatorname{argmin}}\{||y_\mathcal{P} - \tilde{Z}b||_2^2 + b^T B_\lambda b\} \tag{S4}$$

We consider now the linear mixed model (S3) which is equivalent to the problem defined in (S4) with:

(a) $\tilde{\varepsilon} \sim \mathcal{N}(0, \sigma_\varepsilon^2 I_n)$,

(b) $b \sim \mathcal{N}(0, \sigma_\varepsilon^2 B_\lambda^{-1})$.

The maximum likelihood estimators of σ_ε^2 and penalty parameters λ_c, λ_d and λ_R are obtained via maximization of the linear mixed model log-likelihood function written in equation (S7).

Knowing both that

$$y_\mathcal{P} \sim \mathcal{N}(0, \sigma_\varepsilon^2(\tilde{Z} B_\lambda^{-1} \tilde{Z}^T + I_n)) \text{ and } y_\mathcal{P}|b \sim \mathcal{N}(\tilde{Z}b, \sigma_\varepsilon^2 I_n) \tag{S5}$$

and defining

$$\Sigma = \tilde{Z} B_\lambda^{-1} \tilde{Z}^T + I_n, \tag{S6}$$

we can write the log-likelihood function for σ_ε^2, λ_C, λ_D and λ_R as:

$$l(\sigma_\varepsilon^2, \lambda_C, \lambda_D, \lambda_R) = -\frac{1}{2}\left[n\ln(2\pi) + n\ln\sigma_\varepsilon^2 + \ln|\Sigma| + \frac{1}{\sigma_\varepsilon^2} y_\mathcal{P}^T \Sigma^{-1} y_\mathcal{P}\right]. \tag{S7}$$

Firstly, in order to find the maximum likelihood estimator, we search for σ_ε^2 maximizing function l assuming λ_C, λ_D and λ_R as constant numbers:

$$\frac{dl}{d\sigma_\varepsilon^2} = 0 \Leftrightarrow -\frac{1}{2}\left(\frac{n}{\sigma_\varepsilon^2} - \frac{1}{(\sigma_\varepsilon^2)^2} y_\mathcal{P}^T \Sigma^{-1} y_\mathcal{P}\right) = 0 \Leftrightarrow \sigma_\varepsilon^2 = \frac{1}{n} y_\mathcal{P}^T \Sigma^{-1} y_\mathcal{P}.$$

Therefore, taking into account the MLE of σ_ε^2, the likelihood function can be now rewritten in terms of λ_C, λ_D and λ_R:

$$\begin{aligned} l(\lambda_C, \lambda_D, \lambda_R) = &-\frac{1}{2}\left[n\ln(2\pi) + n\ln\left(\frac{1}{n} y_\mathcal{P}^T \Sigma^{-1} y_\mathcal{P}\right) + \ln|\Sigma| + \frac{y_\mathcal{P}^T \Sigma^{-1} y_\mathcal{P}}{\frac{1}{n} y_\mathcal{P}^T \Sigma^{-1} y_\mathcal{P}}\right] = \\ &-\frac{1}{2}\left[n\ln(y_\mathcal{P}^T \Sigma^{-1} y_\mathcal{P}) + \ln|\Sigma| + n + n\ln(2\pi) - n\ln n\right]. \end{aligned}$$

Hence, finding MLE of λ_C, λ_D and λ_R comes down to minimizing:

$$\begin{aligned} \tilde{l}(\lambda_C, \lambda_D, \lambda_R) =& n\ln\left[y_\mathcal{P}^T \Sigma^{-1} y_\mathcal{P}\right] + \ln|\Sigma| = \\ & n\ln\left[y_\mathcal{P}^T (\tilde{Z} B_\lambda^{-1} \tilde{Z}^T + I_n)^{-1} y_\mathcal{P}\right] + \ln|\tilde{Z} B_\lambda^{-1} \tilde{Z}^T + I_n|. \end{aligned} \tag{S8}$$

Using matrix and determinant identities from [1] we can rewrite $\tilde{l}(\lambda_C, \lambda_D, \lambda_R)$ as

$$\begin{aligned} \tilde{l}(\lambda_C, \lambda_D, \lambda_R) =& n\ln\left[y_\mathcal{P}^T (I_n - \tilde{Z}(\tilde{Z}^T\tilde{Z} + B_\lambda)^{-1}\tilde{Z}^T) y_\mathcal{P}\right] + \ln|\tilde{Z} B_\lambda^{-1} \tilde{Z}^T + I_n| \\ =& n\ln\left[||y_\mathcal{P}||_2^2 - y_\mathcal{P}^T \tilde{Z}(B_\lambda + \tilde{Z}^T\tilde{Z})^{-1}\tilde{Z}^T y_\mathcal{P}\right] + \ln|\tilde{Z} B_\lambda^{-1} \tilde{Z}^T + I_n| \\ =& n\ln\left[||y_\mathcal{P}||_2^2 - y_\mathcal{P}^T \tilde{Z}(B_\lambda + \tilde{Z}^T\tilde{Z})^{-1}\tilde{Z}^T y_\mathcal{P}\right] + \ln|B_\lambda + \tilde{Z}^T\tilde{Z}| - \ln|B_\lambda|. \end{aligned} \tag{S9}$$

This is why, like in [2], the maximum likelihood estimators for σ_ε, λ_C, λ_D and λ_R can be written as

$$\begin{cases} \hat{\lambda}^{MLE} = \underset{(\lambda_C,\lambda_D,\lambda_R)}{\text{argmin}}\ \tilde{l}(\lambda_C, \lambda_D, \lambda_R) \\ \hat{\sigma}_\varepsilon^{2MLE} = \frac{1}{n} y_\mathcal{P}^T (\tilde{Z} B_\lambda \tilde{Z}^T + I_n)^{-1} y_\mathcal{P} \end{cases}. \tag{S10}$$

2 SUPPLEMENTARY FIGURES

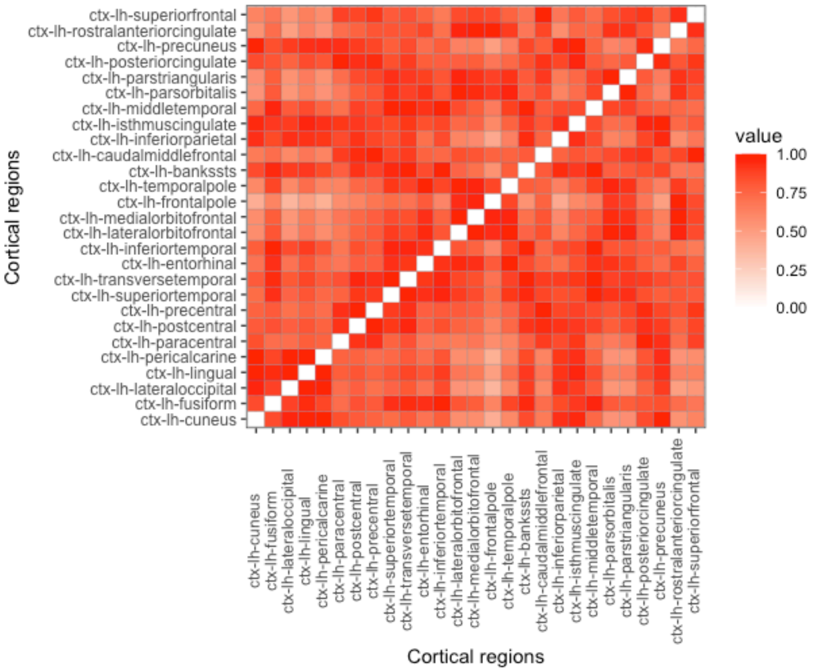

Figure S1. Proximity matrix displayed for the left hemisphere, Euclidean distance and parameter $h = 1$.

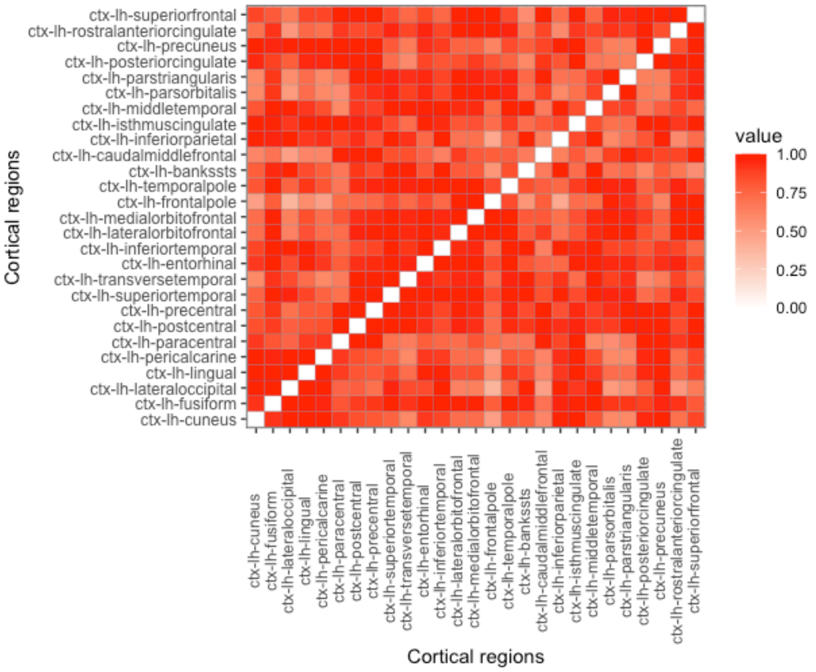

Figure S2. Proximity matrix displayed for the left hemisphere, geodesic distance and parameter $h = 1$.

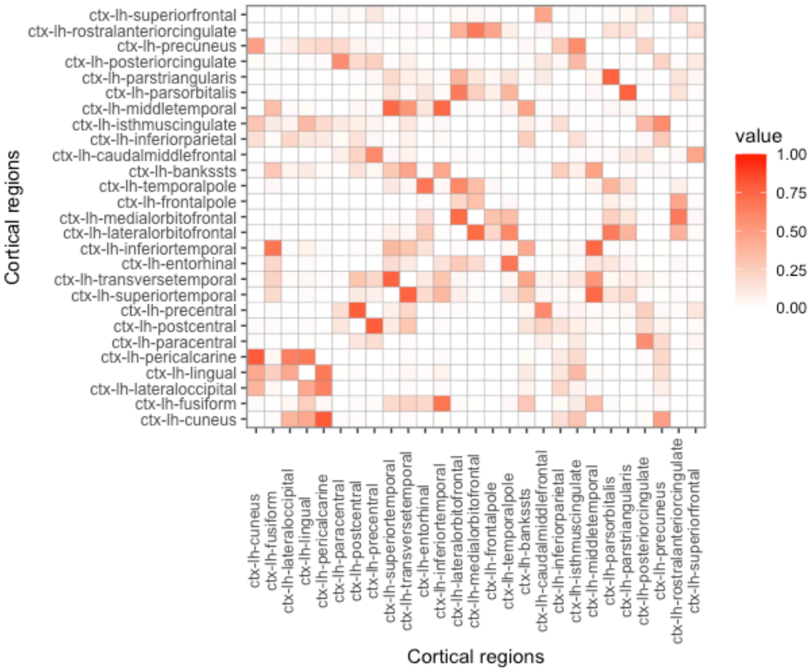

Figure S3. Proximity matrix displayed for the left hemisphere, Euclidean distance and parameter $h = 25$.

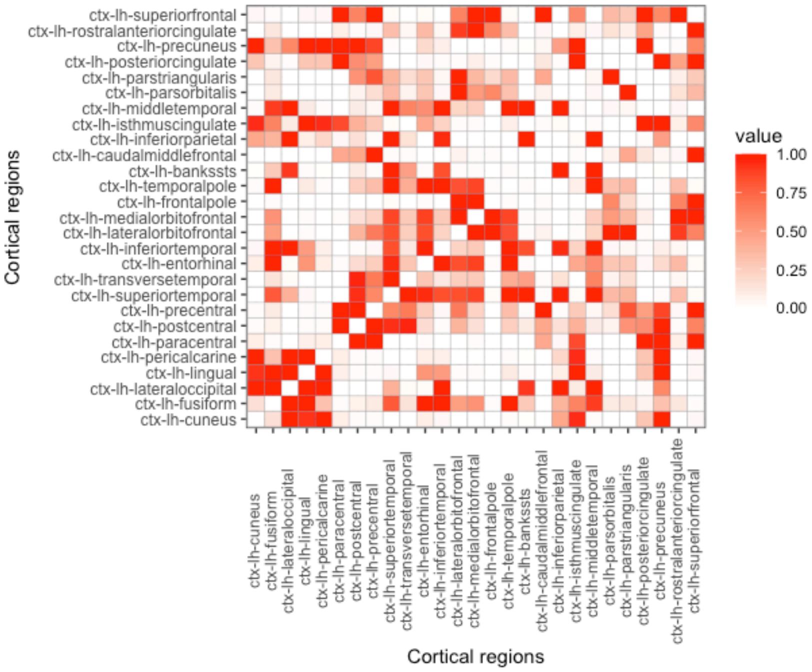

Figure S4. Proximity matrix displayed for the left hemisphere, geodesic distance and parameter $h = 25$.

REFERENCES

[1]Eugene Demidenko. *Mixed models: theory and applications with R*. John Wiley & Sons, 2013.

[2]Marta Karas, Damian Brzyski, Mario Dzemidzic, Joaquin Goni, David A. Kareken, Timothy W. Randolph, and Jaroslaw Harezlak. Brain connectivity-informed regularization methods for regression. *Statistics in biosciences*, 11(1):47–90, 2019.

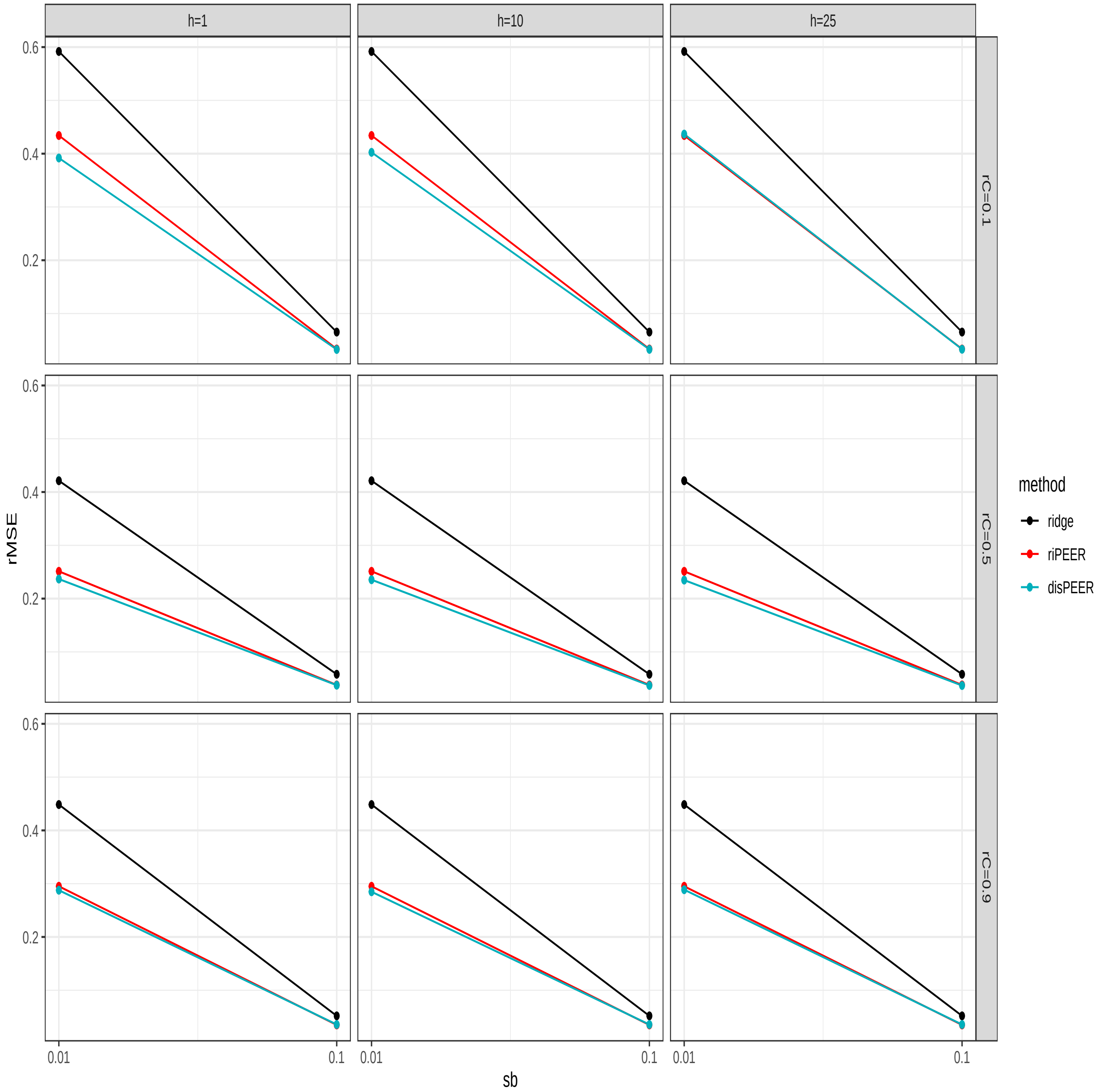

Figure S5. Plots of *rMSE* for b estimates for different connectivity information configurations $r_C \in \{0.1, 0.5, 0.9\}$ with $\sigma_b \in \{0.01, 0.1\}$, $\sigma_\varepsilon = 1$ and $h = 5$ with different proximity parameter $\hat{h} \in \{1, 10, 25\}$ used within the estimation (**left hemisphere** and **Euclidean distance**).

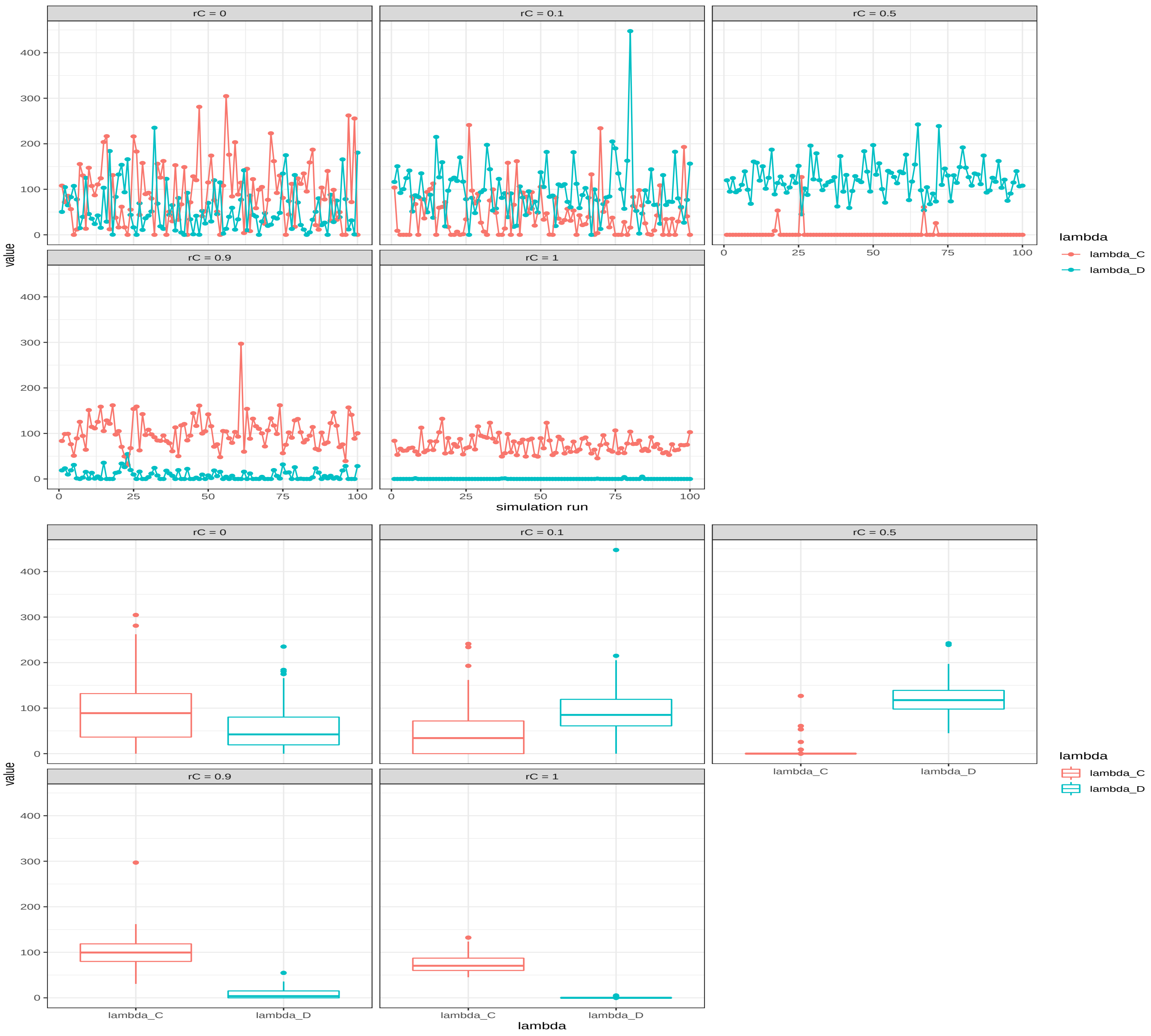

Figure S6. Path plots and boxplots of penalty parameters λ_C and λ_D for *disPEER* with different connectivity information configurations $r_C \in \{0, 0.1, 0.5, 0.9, 1\}$ with $\sigma_b = 0.01$, $\sigma_\varepsilon = 1$ and $h = 5$ (**left hemisphere** and **Euclidean distance**).

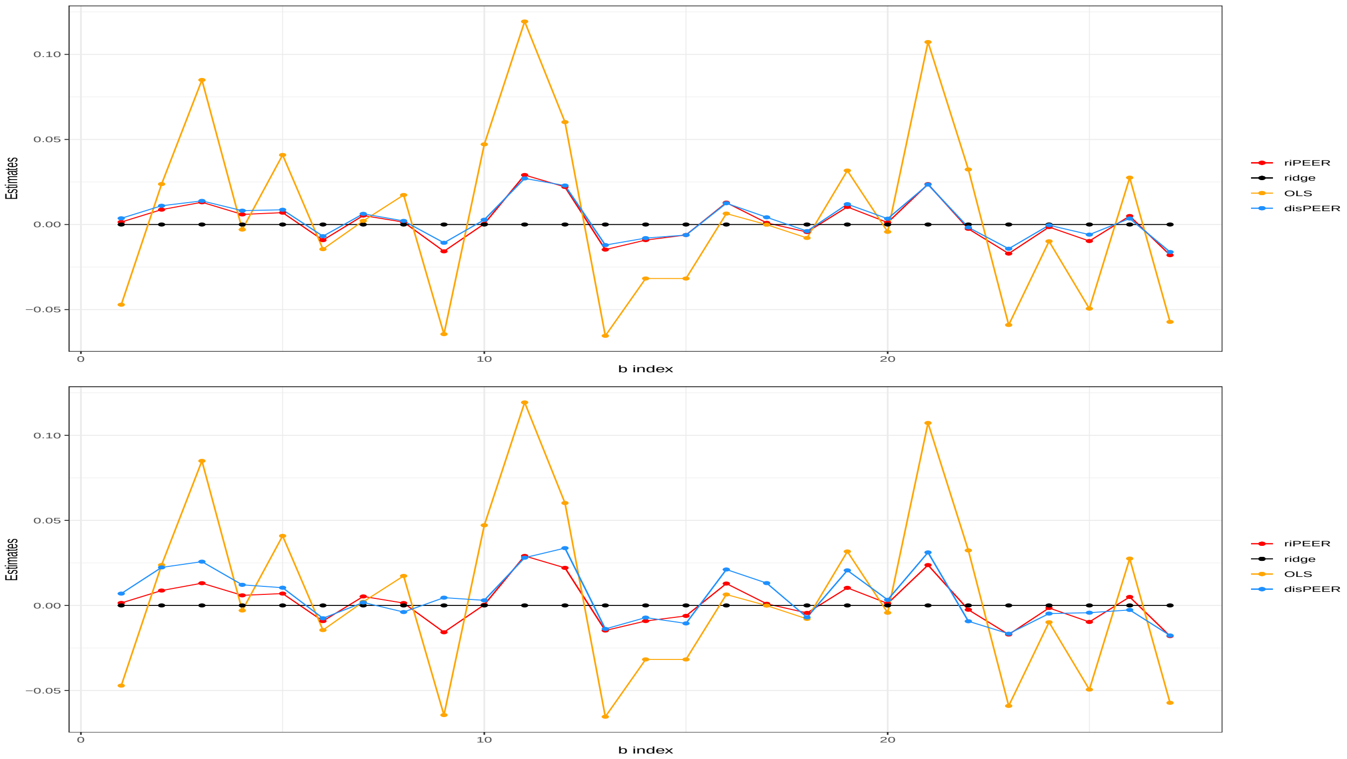

Figure S7. Plots of b estimations within OLS, ridge, *riPEER* and *disPEER* modeling Language/Vocabulary Comprehension cognitive function for Euclidean and geodesic distance (upper and lower, respectively) and the left hemisphere.

Table S1. *rMSE* (median) results for *ridge*, *riPEER* and *disPEER* methods with $h = 5$ and $h = 100$ for Euclidean and geodesic distances respectively for $n = 100$, $\sigma_b^2 \in \{0.1, 0.01\}$ and $\sigma_\varepsilon^2 \in \{1, 5\}$. Last column denotes percentage of *rMSE* decrease for *disPEER* in respect of *riPEER* ("-" indicates increase).

rMSE results (100 loops)						
r_C	$\sigma_b^2/\sigma_\varepsilon^2$	distance	*Ridge*	*riPEER*	*disPEER*	*rMSE* decrease [%]
0.1	0.100	*Euc.*	0.1507	0.1465	0.1287	13.8
0.1	0.010	*Euc.*	0.7824	0.9121	1.0020	-9.9
0.1	0.020	*Euc.*	0.5413	0.4876	0.3992	22.1
0.1	0.002	*Euc.*	0.9895	1.6140	1.8990	-17.7
0.5	0.100	*Euc.*	0.2131	0.1202	0.1197	0.4
0.5	0.010	*Euc.*	0.9287	0.7998	0.8724	-9.1
0.5	0.020	*Euc.*	0.6658	0.3042	0.3167	-4.1
0.5	0.002	*Euc.*	0.9933	1.8110	1.8680	-3.1
0.9	0.100	*Euc.*	0.1621	0.0805	0.0797	1.0
0.9	0.010	*Euc.*	0.8096	0.3990	0.4225	-5.9
0.9	0.020	*Euc.*	0.7477	0.2572	0.2659	3.4
0.9	0.002	*Euc.*	0.9912	0.9320	1.0080	-8.2
0.1	0.100	*geod.*	0.2131	0.2478	0.2359	5.0
0.1	0.010	*geod.*	0.9191	1.1920	1.3520	-13.4
0.1	0.020	*geod.*	0.7085	0.8603	0.8689	-1.0
0.1	0.002	*geod.*	0.9955	2.1610	2.4790	14.7
0.5	0.100	*geod.*	0.2081	0.1904	0.1957	-2.8
0.5	0.010	*geod.*	0.9484	0.7669	0.7580	1.2
0.5	0.020	*geod.*	0.7922	0.6525	0.6690	-2.5
0.5	0.002	*geod.*	0.9977	1.5900	1.8520	-16.5
0.9	0.100	*geod.*	0.2148	0.2108	0.2097	0.5
0.9	0.010	*geod.*	0.8998	0.6241	0.6369	-2.0
0.9	0.020	*geod.*	0.5391	0.7104	0.7139	0.5
0.9	0.002	*geod.*	0.9948	1.3620	1.4650	-7.6

Table S2. *rMSE* (median) results for *ridge*, *riPEER* and *disPEER* methods with $h = 5$ and $h = 100$ for Euclidean and geodesic distances respectively for n = 200, $\sigma^2 \in_b \{0.1, 0.01\}$ and $\sigma_\varepsilon^2 \in \{1, 5\}$. Last column denotes percentage of *rMSE* decrease for *disPEER* in respect of *riPEER* ("-" indicates increase).

rMSE results (100 loops)						
r_C	$\sigma_b^2/\sigma_\varepsilon^2$	distance	*Ridge*	*riPEER*	*disPEER*	*rMSE*
decrease [%]						
0.1	0.100	*Euc.*	0.0857	0.0712	0.0718	-0.8
0.1	0.010	*Euc.*	0.5399	0.4594	0.4707	-2.5
0.1	0.020	*Euc.*	0.3548	0.2949	0.3339	-13.2
0.1	0.002	*Euc.*	0.9852	0.9594	1.0790	-12.5
0.5	0.100	*Euc.*	0.0826	0.0585	0.0587	-0.3
0.5	0.010	*Euc.*	0.6573	0.6377	0.6273	1.7
0.5	0.020	*Euc.*	0.3524	0.1863	0.1907	-2.4
0.5	0.002	*Euc.*	0.9908	1.1530	1.1070	4.2
0.9	0.100	*Euc.*	0.0632	0.0451	0.0452	-0.2
0.9	0.010	*Euc.*	0.5189	0.1943	0.1894	2.6
0.9	0.020	*Euc.*	0.2944	0.1193	0.1187	0.5
0.9	0.002	*Euc.*	0.9704	0.5295	0.5949	-12.4
0.1	0.100	*geod.*	0.1240	0.1102	0.1094	0.7
0.1	0.010	*geod.*	0.7566	0.7495	0.7702	-2.8
0.1	0.020	*geod.*	0.4568	0.4924	0.4768	4.2
0.1	0.002	*geod.*	0.9919	1.5180	1.6640	-9.6
0.5	0.100	*geod.*	0.1160	0.0848	0.0844	0.4
0.5	0.010	*geod.*	0.7524	0.6256	0.5820	7.5
0.5	0.020	*geod.*	0.4928	0.3085	0.3172	-2.8
0.5	0.002	*geod.*	0.9916	1.1160	1.1190	-0.3
0.9	0.100	*geod.*	0.1143	0.0953	0.0958	-0.5
0.9	0.010	*geod.*	0.7433	0.3322	0.3327	-0.2
0.9	0.020	*geod.*	0.4274	0.4016	0.4010	0.1
0.9	0.002	*geod.*	0.9964	0.9356	0.9924	-6.1
